# Phylogeographic Analysis Elucidates the Influence of the Ice Ages on the Disjunct Distribution of Relict Dragonflies in Asia

**DOI:** 10.1371/journal.pone.0038132

**Published:** 2012-05-30

**Authors:** Sebastian Büsse, Philipp von Grumbkow, Susanne Hummel, Deep Narayan Shah, Ram Devi Tachamo Shah, Jingke Li, Xueping Zhang, Kazunori Yoshizawa, Sonja Wedmann, Thomas Hörnschemeyer

**Affiliations:** 1 Johann-Friedrich-Blumenbach-Institute of Zoology and Anthropology, Department of Morphology, Systematics and Evolutionary Biology, Georg-August-University Göttingen, Göttingen, Germany; 2 Johann-Friedrich-Blumenbach-Institute of Zoology and Anthropology, Department of Historical Anthropology and Human Ecology, Georg-August-University Göttingen, Göttingen, Germany; 3 Department of River Ecology and Conservation, Senckenberg Research Institutes and Natural History Museums, Gelnhausen, Germany; 4 Hindu Kush Himalayan Benthological Society, Kathmandu, Nepal; 5 Vientiane, Laos; 6 Key Laboratory of Remote Sensing Monitoring of Geographic Environment, College of Heilongjiang Province, Harbin Normal University, Harbin, China; 7 Systematic Entomology, Graduate School of Agriculture, Hokkaido University Sapporo, Japan; 8 Senckenberg Research Institutes and Natural History Museums, Research Station Messel Pit, Messel, Germany; American Museum of Natural History, United States of America

## Abstract

Unusual biogeographic patterns of closely related groups reflect events in the past, and molecular analyses can help to elucidate these events. While ample research on the origin of disjunct distributions of different organism groups in the Western Paleartic has been conducted, such studies are rare for Eastern Palearctic organisms. In this paper we present a phylogeographic analysis of the disjunct distribution pattern of the extant species of the strongly cool-adapted *Epiophlebia* dragonflies from Asia. We investigated sequences of the usually more conserved 18 S rDNA and 28 S rDNA genes and the more variable sequences of ITS1, ITS2 and CO2 of all three currently recognised *Epiophlebia* species and of a sample of other odonatan species. In all genes investigated the degrees of similarity between species of *Epiophlebia* are very high and resemble those otherwise found between different populations of the same species in Odonata. This indicates that substantial gene transfer between these populations occurred in the comparatively recent past. Our analyses imply a wide distribution of the ancestor of extant *Epiophlebia* in Southeast Asia during the last ice age, when suitable habitats were more common. During the following warming phase, its range contracted, resulting in the current disjunct distribution. Given the strong sensitivity of these species to climatic parameters, the current trend to increasing global temperatures will further reduce acceptable habitats and seriously threaten the existences of these last representatives of an ancient group of Odonata.

## Introduction

Disjunct biogeographic patterns with closely related organisms occurring in widely separated areas are puzzling. Such disjunct distributions reflect historical events, and a major goal of research is to deduce which events were responsible for the given distribution pattern. Disjunct ranges of closely related taxa can come into existence either by tectonics, by dispersal or by intervening extinction [1]. For European mountain ranges it has already been shown that today’s disjunct distribution patterns of many cold-adapted species are post ice age relicts [2]. However, the study of disjunct species and their phylogeography in other areas such as e.g. the Himalayas and Southeast Asia is still in its infancy.

In this paper we illuminate the disjunct distribution pattern of *Epiophlebia,* a unique dragonfly from Asia.

Traditionally, the Odonata have been divided into Anisoptera (dragonflies), Zygoptera (damselflies) and “Anisozygoptera”. Besides the extant species of *Epiophlebia* the “Anisozygoptera” comprised mainly Jurassic fossils [3] until it was shown that “Anisozygoptera” are not monophyletic [3]–[5]. Presently, *Epiophlebia* is considered to be the most basal sistergroup of the Anisoptera, with several extinct lineages nested in between [4]–[7]. A close relationship of Anisoptera and *Epiophlebia* has also been corroborated by several molecular analyses and the term Epiprocta has been introduced for this grouping [8]–[11].

The species of *Epiophlebia* have been considered as “living fossils” [12] because they display features of both damselflies (Zygoptera) and dragonflies (Anisoptera). In their general body outline they resemble dragonflies, but as in damselflies their fore- and hind wings are similarly shaped and petiolate [12]. Like dragonflies, the larvae use a rectal chamber for respiration, but jet propulsion, which is typical of dragonflies, was never observed [13].

So far it was assumed that there are three extant species of *Epiophlebia* but there were doubts on the species differentiation between *E. superstes* (Sélys, 1889) and *E. laidlawi* Tillyard, 1921 from early on [12], [14]–[17]. Recently, a third species *E. sinensis* Li and Nel, 2011 was described from China [18].

While the Japanese *E. superstes* was thoroughly described by Asahina [12], [16], *E. laidlawi*, and *E. sinensis* are only poorly known. The morphological discrimination between species is based only on the following features [16], [17], [19]: adults of *E. laidlawi* differ from *E. superstes* in larger size and brownish body coloration, parts of the male genitalia differ slightly in shape, the apical process of the eighth sternite is less developed in the female of *E. laidlawi*, and the wings are slightly longer [17]. For the larvae of *E. laidlawi* the following differences to *E. superstes* have been descibed [16: p. 445]: slightly larger body size; length and form of the third antennal segment; form of the antero-lateral angle of the pronotum, form of the fore femur, development of the lateral spines of abdominal segments 7 to 9, and differences in the shape of the epiproct.

*E. sinensis* was described on the base of two adult male specimens, which differ from the other species in the hairiness of the epiproct and in the colouration of the abdomen [18].

Nowadays, the distribution of *Epiophlebia* is disjunct. *E. superstes* is restricted to large areas of Japan [12], [13], [20], *E. laidlaw*i is found only in the Himalayas [14], [16], [19], [21] and the recently described *E.* *sinensis* adds another dot in north-east China to the disjunct pattern. This distribution is due to very specific habitat requirements. *Epiophlebia* prefers cold mountain streams with temperatures of about 4 to 5°C in winter and about 16–17°C in summer (data published for *E. superstes* [13]) and altitudes between 1,300 to approximately 3,000 m (for *E. laidlawi* [21]). The recently described *E. sinensis* also fits into this pattern as it was collected in the vicinity of a mountain stream, however, at an elevation of not more than 500 m [18]. In a recent discussion the biogeography of *E. superstes* and *E. laidlawi* [21] Epiophlebiidae and the extinct closely related Stenophlebiidae are considered as part of an “archeo-palaearctic dragonfly fauna” that formed in the Mesozoic and during the Tertiary was intermingled with oriental faunal elements. The extant *Epiophlebia* is considered as belonging to this ancient fauna, parts of which survived on the Japanese islands, in the Himalayas and in China [18], [21].

The present study aims at clarifying the phylogenetic relationships and the biogeographic history of *E. superstes*, *E. laidlawi* and *E.* *sinensis* from a genetic point of view. Since DNA sequences so far were only available for *E. superstes* [8], [22], [23] we had to acquire additional sequence data for specimens from the other species and from different populations. The specimens available had originally been preserved for morphological and faunistic research, applying preservatives containing, among others, formaldehyde. Thus, we applied techniques used for analysis of degraded DNA [24] to get sequences of sufficient length. To achieve a good resolution of relationships on all taxonomic levels we investigated conserved genes as well as more variable regions [8], [25], [26].

Here we present the unexpected homogeneity of DNA-sequences of the supposed species *E. laidlawi*, *E. superstes* and *E.* *sinensis*. In the light of our new data, the extant disjunct distribution of *Epiophlebia* is explained in a distinctly different scenario than those suggested by [21] or [18].

## Results

The specimens of *Epiophlebia* initially were not collected and preserved for subsequent DNA analysis. Thus, amplification and sequencing turned out to be problematic. However, for all targeted genes it was possible to acquire sections of up to 300 bp (Table S1). These were positioned in such a way, that phylogenetic relevant sequences could be expected while allowing straightforward alignment throughout at least the Odonata.

For *E. superstes* and *E. laidlawi* sequences of 18 S, and 28 S rDNA, ITS1, ITS2 and CO2 genes were analysed as well as CO1 for *E. superstes*. For *E. laidlawi* it was not possible to get a sequence for CO1. Due to contamination with other odonatan DNA we could only acquire the ITS sequences for *E. sinensis*, because our primers are highly specific for these genes.

All sequences show an extreme degree of similarity between all *Epiophlebia* species.

### DNA Analysis

Sequences of *E. superstes* from [8] were used as reference. The sequences of 18 S rRNA (240 bp) and 28 S rRNA genes (1∶191 bp; 2∶267 bp; 3∶251 bp; 4∶293 bp) did not show any differences between *E. laidlawi* (specimens NATR3 & NA01) and *E. superstes* (our sequences and FN356086, EU424328). For CO2 (265 bp) a single difference at position 368 relative to the *E.* *superstes* reference sequence (EU055421) was found in all specimens including the Japanese control specimen (Figure S1).

Likewise, the fragment of ITS2 (265 bp) shows one deletion of G at position 2613 (relative to reference sequence FN356086) in all specimens investigated. Additionally, a maximum of three more differences were found in one of the *E. laidlawi* specimens (NA01) the other specimen (NATR03) showed only one difference and in *E. sinensis* there are two (Figure S2).

**Figure 1:**
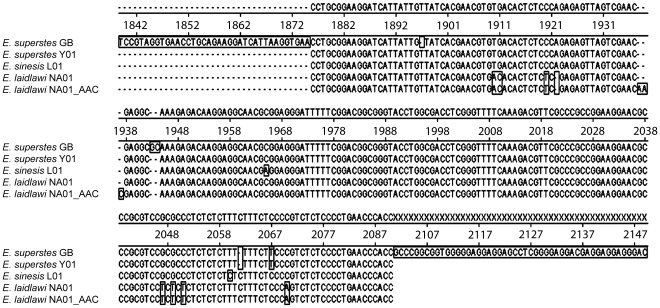
Alignment of ITS1 sequences from different specimens of *Epiophlebia* species. *E. superstes*_GB = reference sequence from GenBank.

In ITS1 (215 bp) two deletions (one G at position 1943 and one C at position 1944, relative to the *E. superstes* reference sequence from GenBank) are common to all specimens investigated. Additionally, ITS1 shows a maximum of 11 differences in *E.* *laidlawi* and seven in *E. sinensis*. In specimen NA01 of *E.* *laidlawi* also a heterozygous duplication of AAC at position 1935 of ITS1 was detected (Figure 1).

### Phylogenetic Analysis

The complete dataset for phylogenetic analysis contained 17 taxa including a representative of Zygentoma as outgroup, two specimens of both *Epiophlebia laidlawi* and *E. superstes* and one specimen of *E. sinensis*. For each gene one sequence for *E. superstes* was taken from GenBank (Table S1). The 4799 characters were composed of 537 positions from the CO2 gene, 1700 positions from 18 S, 2157 positions from 28 S, 231 nucleotides from ITS2 and 173 nucleotides from ITS1. For some of the taxa in the matrix sequences from different species, or specimens of uncertain species determination, of a certain genus had to be combined into a chimeric sequence. This is true for Zygentoma, Calopterygidae, Lestidae, Gomphidae, Cordulegasteridae, Aeshnidae, Corduliidae and Coenagrionidae (Table S1). Alignment of ITS sequences turned out to be problematic due to their high variability. Eventually, an alignment was accepted, produced with the online version of MAFFT [27] with standard parameters, except for the scoring matrix set to “20PAM/k = 2” and the offset value set to 0.1. Highly variable sections were removed from the dataset.

Since it was not possible to obtain a sequence of CO1 for *E.* *laidlawi* or *E. sinensis*, this gene was not used in the phylogenetic analysis.

Data were formatted as mixed dataset for analysis with MrBayes 3.1.2. For each data partition a model was selected with MrModeltest 2.3: for CO2, 18 S and 28 S GTR+I+G was used, for ITS2 F81+G and for ITS1 JC+G. A NEXUS file with alignments and all parameters used can be found in Dataset S1.

**Figure 2:**
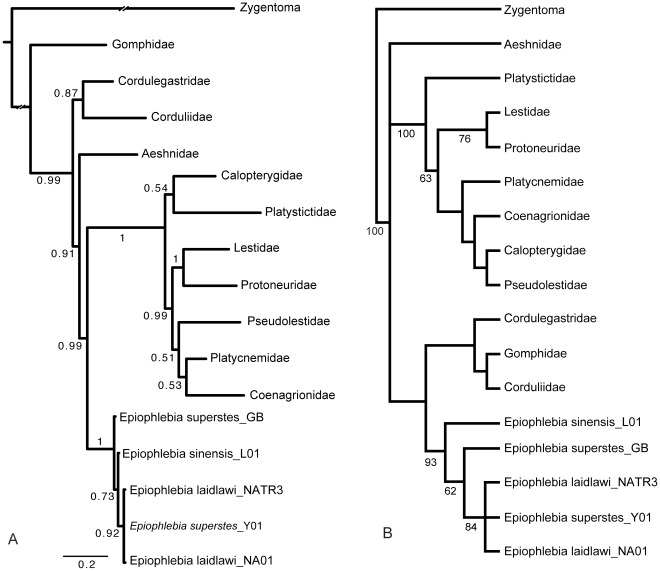
Results of phylogenetic analyses. (A) Phylogram of bayesian analysis of full dataset (see text). Numbers indicate posterior probability for respective branches. (B) Strict consensus of three trees from maximum likelihood analysis. Numbers indicate bootstrap values. Where these values are missing the respective node collapsed in the bootstrap analysis.

Bayesian analysis over four million generations produced the phylogram shown in Figure 2A.

For the objective of this investigation the relationships of the *Epiophlebia* specimens are most important. These specimens do not appear in a sistergroup relationship with the other specimen of the same species. This result is reproduced when analysing the dataset with the maximum likelihood algorithm (ML) (SYM+I+G used for the complete dataset, Figure 2B) or under maximum parsimony (hsearch, addseq = random, nreps = 100; both in PAUP*). With respect to the relationships of the *Epiophlebia* specimens also phylogenetic analyses based only on single genes reproduced the same arrangement.

The position of *Epiophlebia* as sistergroup of Zygoptera and the non-monophyly of Anisoptera are remarkable. These results contradict the widely accepted hypothesis of a monophyletic Anisoptera and a recently published hypothesis based on more comprehensive datasets proposing a sistergroup relationship between *Epiophlebia* and Anisoptera [8]–[11]. However, in the maximum likelihood analysis (Figure 2B) a sistergroup relationship of *Epiophlebia* and a clade containing Anisoptera with the exception of Aeshnidae is recovered.

### Morphology

The external morphology of several larval instars of *E. superstes* and *E. laidlawi* was compared by [16]. He concluded that they might well be two separate species based on six differing characters. Four of these characters could not be confirmed by our morphological investigation of larvae of both species. Two characters, the lateral posterior corners of the abdominal segments 7 to 9, and the shape of the epiproct consistently show slight differences between these supposed two species. Larvae of *E.* *sinensis* were not available for investigation.

Differences in the adults are restricted mainly to coloration. Himalayan specimens seem to be more brownish than the black Japanese specimens and the Chinese ones have a reddish hint in the posterior area of the abdomen. Furthermore, there are small differences in the arrangement of setae and in the shape of the male genitalia [12], [14]–[18], [28].

In total, the visible differences between specimens from the different regions allow identifying where a specimen was found. However, a general problem is the small number of known specimens from the Himalayas and from China. Thus the variability of morphological features cannot be determined.

## Discussion

When assembling and aligning the sequences from *Epiophlebia superstes*, *E. laidlawi* and *E. sinensis*, it quickly became obvious that there are only very few differences between these supposed species.

In order to be able to adequately rate this observation, we compared sequences of several different closely related odonatan species as well as specimens of the same species from different populations (Table 1). Between representatives of different species there usually are significantly more differences over similar sequence length in the same area of a gene than are present between the specimens of *Epiophlebia*. In most species the number of intraspecific differences (e.g. in CO2 of *Ischnura asiatica*) is higher than the number of differences found between species of *Epiophlebia*. Even the usually variable partial 28 S sequences show no polymorphisms between the investigated specimens. We were only able to locate the more or less identical region of ITS from GenBank for other Odonata species. Unfortunately, for 18 S, 28 S and CO2 we were not able to locate the same sections in enough specimens of closely related species or of different specimens of the same species for such a comparison. However, due to the used sequencing method we identify the most variable regions of 18 S and 28 S and even those show no differences.

**Table 1:** Inter- and intra-specific variation in different species of Odonata for the sequences investigated.

Sequence	Species/Populations	Accession No.	No. of mutations/length of sequence	Max. no. of mutations/length of sequence between *Epiophlebia* specimens
18S	*Enallagma parvum/E. nigridorsum*	AJ420939/AJ420938	3/1856	0/240
28S	*Orthetrum albistylum/O. triangulare*	AB127411/AB127410	6/603	0/1002
CO2	*Lestes sponsa/L. temporalis*	AB446428/AB446429	28/282	0–1/282
CO2	*Ischnura asiatica*	AB446399/AB446400/AB446401	4/283	
ITS2	*Calopteryx splendens/C. maculata*	AJ308363/AJ459198	25/253	4/265
ITS2	*Calopteryx haemorrhoidalis*:Italy/Morocco	AJ308348/AJ308347	3/213	
ITS1	*Anax panybeus*/*A. guttatus*	AB601902/AB601901	11/261	11/215*
ITS1	*Cordulia aenea*	AY274516/AY274535/AY274537/AY274539	14/283	

* a total of 11 polymorphic nucleotide positions which probably arose from only two mutation events.

The occurrence of a heterogeneous insertion of three base pairs as well as the clustering of polymorphisms in ITS1 in the *E. laidlawi* specimens indicates that this region is comparatively unstable. Slippage events might occur more often than in other regions and it is likely that the observed polymorphisms arose from two slippage events rather than one-by-one through point mutations. Thus, we propose that the 11 differences observed between *E.* *laidlawi* and *E. superstes* can be traced back to only four different events.

Even though there are so few variations between the specimens investigated we even observed a few different positions between the *E. superstes* sequences from GenBank that was used for comparison and the newly produced sequences. The specimens that were sequenced for the GenBank data are from a population in the vicinity of Tokyo, whereas our specimens are from Hokkaido in the far north of Japan. Therefore the observed differences might just reflect the sequences’ origin form different populations and also indicate that there is not very much gene exchange between these populations.

The comparisons of sequences of different populations and of closely related species (Table 1) reveal that the interspecific variability in DNA sequences within different populations of Odonata can be much higher than between specimens of *Epiophlebia*. In total this suggests that gene transfer between its species and populations took place in the not so distant past.

### Phylogenetic Analysis

Despite the differences in the general topology of trees generated with maximum parsimony, maximum likelihood and bayesian algorithms the specimens of *Epiophlebia* are always grouped in one well-supported clade but never appear in the arrangement of the currently recognized species. This arrangement, with specimens of *Epiophlebia* freely mixed on the tree, is independent of the composition of the dataset and of the algorithm applied. Even the fairly variable CO- and ITS-sequences produce this result when analysed separately. These results challenge the assumption of three separate extant species of *Epiophlebia*. Further investigations especially of the morphology should be done to clarify the variability of discriminating features and the taxonomic status of the three species.

Further results of the present phylogenetic analysis stating a non-monophyletic Anisoptera and a sistergroup relationship of *Epiophlebia* and Zygoptera are remarkable. Taxa for the analysis were originally selected to represent major clades from the phylogenetic system of Odonata as reconstructed in [8] and we expected to recover its general topology. However, our taxon sample is not as comprehensive as in any of the investigations that recently confirmed monophyletic Anisoptera with a sistergroup *Epiophlebia* [6], [8], [10], [11]. Therefore, the differing topology found in the present analysis may well be attributed to effects of the composition of taxa in the dataset. Monophyletic Anisoptera as well as its sistergroup relationship with *Epiophlebia* are also supported by morphological characters found in extant as well as in fossil Odonata [3], [29], [30]. These relationships seem to be more probable than the topology found in our analyses, since our dataset was not compiled to be especially informative in respects of the relationships of high-level taxa of Odonata. However, since our results are reproducible with different algorithms and from different combinations of sequence data, they should be understood as a strong indication that higher level phylogenetic relationships within Odonata might not yet be finally resolved.

### Biogeographic History of *Epiophlebia*

Firm biogeographic connections between the area of the present Himalayas, the Asian mainland and of Japan in former times are well documented by the Sino-Japanese floristic region [31]. However, since when an effective isolation of the Asian mainland and the Japanese populations is established, can only be estimated.

**Figure 3:**
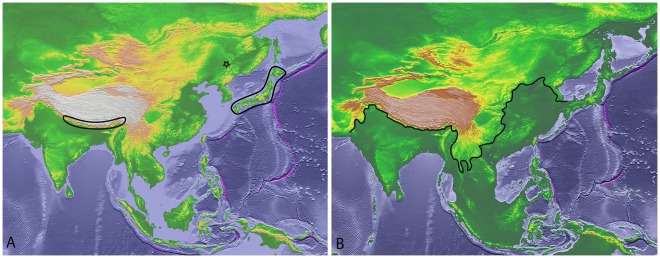
Distribution of *Epiophlebia*. (A) Known present distribution, the star marks the type locality of *E. sinensis* Li & Nel, 2011. (B) Approximate coastline during the Würm glacial period with land bridges between Japan and the mainland. The black line marks a possible northern boundary of the range of *Epiophlebia* during this time. (Modified from [47]).

Presently Japan is separated from the mainland by sea-straits with depths of ca. 55 m north of Hokkaido and ca. 130 m between the southern island Kyushu and Korea [32]. The last substantial land bridge to Hokkaido was present during the Würm sea level lowering approximately 20,000 years BP [33]. According to some authors [32], [34], [35] a land bridge also existed between Kyushu and the Korean peninsula during this time (Figure 3). Assuming that during this period was the most recent possibility for genetic exchange between the Japanese and the mainland populations of *Epiophlebia,* it also follows that their distribution was significantly different from the present one.

*Epiophlebia* larvae apparently are very stenoecious and inhabit only cool headwaters of streams. During the last glacial maximum suitable environmental conditions probably were present throughout the lowlands south, southeast and east of the Himalayas. The area with adequate environmental conditions probably reached at least northwards to the connection between the southern Japanese island Kyushu and the mainland (Figure 3B). When the temperatures rose again at the end of the last ice age *Epiophlebia* retreated to the cooler higher areas. Consequently, the extant populations of *Epiophlebia* are small relicts of a formerly much larger and wider distributed population.

Previously the extant distribution of *Epiophlebia* was dated back to the Jurassic when Pangäa broke apart [18], [21]. This might be true for the general Asiatic distribution of the ancestors of *Epiophlebia*. However, our data show that the extant species and most likely the genus are very much younger. Therefore, the interpretation that even the species go back to Jurassic time [21] can not be maintained.

Current climatic development threatens the existence of these populations [36]. With globally rising temperatures suitable habitats for species like *Epiophlebia* with such narrow tolerances for environmental parameters are in great danger of extinction.

### Status of *Epiophlebia* Species

In the light of the extreme similarity of sequences in *Epiophlebia* specimens from Japan and Nepal, the observed morphological differences between their adults as well as their larvae tend to appear as minor local variations. In summary, this indicates that probably the Himalayan and the Japanese populations are in fact representatives of a single biological species. However, with the current climatic and topographic situation the chances of future genetic exchange between these populations are bleak. So, even if at present the different populations just represent one biological species, the probability that they are just on the verge of becoming “real” species is very high.

One might even assume that the populations in the different valleys of the Himalayas are separated from one another by the high mountain ranges as effectively as any of them is separated from the Japanese population by sheer distance [19]. So, perhaps even here there is a certain probability for the future formation of individual species.

For *E. sinensis* the situation is not very different. Only the amount of available data for this species is much worse than for the Japanese and the Himalayan populations. No larvae are known so far and sequence data are also sparse. Furthermore, the available information on the morphology is based on no more than two male specimens [18]. So, we know nothing about morphological variability. The available sequence data are, however, as similar to those of *E. superstes* as those from *E. laidlawi* are. If our biogeographic interpretations are correct, than it even seems to be probable that genetic exchange between *E. superstes* and *E. sinensis* was possible until a more recent time than between *E. superstes* and *E. laidlawi*. Thus, *E. sinensis* might also be just another population of this single species of *Epiophlebia*.

### Mutation Rates of Different DNA-sequences of *Epiophlebia*

The analyses of the different sequences indicate a low variability even in quickly evolving parts of the genome as in the ITS genes. This implies that the range expansion of *Epiophlebia* during the last glacial maximum was very quick, because these circumstances enhance low genetic diversity in a population [37].

On the other hand, this might present an opportunity to estimate mutation rates backwards, at least for the highly variable sections of the DNA. Assuming that the genome was very homogeneous after range expansion during the last glacial maximum about 20,000 years ago, the substitution rate can be inferred from the number of differences in the sequences that we were able to acquire for *Epiophlebia*:

For ITS1 a rate of 4.65×10^−7^ to 2.56×10^−6^ substitutions per site per year can be deduced, for ITS2 there are 5.19×10^−7^, and for CO2 1.89×10^−7^ substitutions per site per year. Compared to such rates in other organisms [38], [39] the rates for *Epiophlebia* are clearly among the higher values. Nevertheless, these figures do not contradict the assumption of a comparatively recent separation of the *Epiophlebia* populations.

## Materials and Methods

Eleven larvae of *Epiophlebia superstes* (Sélys, 1889) of different instars were collected in 2010 in Hokkaido, Japan, fixed and stored in 80% ethanol.

Twelve larvae of different instars of *E. laidlawi* Tillyard, 1921 were collected in 2008 and 2009 in Nepal, fixed in 4% formaldehyde and stored in 70% ethanol. Specimens are stored in the collection of the Hindu Kush Himalayan Benthological Society, Nepal.

Two adults of *E. sinensis* Li and Nel, 2011 were collected in 2011 in Heilongjiang province, China as described in [18]. Two femora of these specimens were available for sequencing.

Larvae of *Coenagrion* spec. were collected in the botanical garden of the Georg-August-University in Göttingen, Germany; fixed in FAE and stored in 70% ethanol (to simulate the conditions of preservation of the other specimens).

For detailed morphological investigation tomography data of four larvae of *E. laidlawi* and three specimens of *E. superstes* were acquired at the Swiss Light Source synchrotron (SLS, Viligen, Switzerland, proposal no. 20100088 by TH) and with a v|tome|x s X-ray scanner (GE Sensing & Inspection Technologies GmbH phoenixjx-ray) at the Palaeontological Institut at University Bonn (Germany).

### Contamination Prevention

The DNA analysis was carried out under strict safety conditions [24], such as separation of pre- and post-PCR laboratories and the use of disposable protective clothing, glassware, and disposable gloves. All experiments took place with disposable laboratory ware, such as pipette tips and cups, while workbenches and other laboratory equipment were cleaned with detergents (Alconox™ Detergent, Aldrich, Germany), bi-distilled water, and ethanol before use for each sample to avoid cross-contamination. In accordance with the recommendations of [40], all disposable ware and solutions, buffers, and MgCl_2_ were irradiated with ultraviolet light at a short distance employing aluminum foil coating. Negative PCR and extraction controls were employed.

### DNA Extraction

Before DNA extraction the guts of the specimens were removed to avoid possible contamination with foreign DNA. Only thorax and leg muscles were used for the analyses.

For cell lysis, 200 µl ATL buffer (Qiagen, Germany) was added to 10–20 mg of tissue. The mixture was homogenized in a TissueLyser (Qiagen) at 30 Hz for 60 s using a 5 mm steel ball. After removal of the ball, 30 µl of Proteinase K (20 mg/ml) was added to the solution and incubated at 56°C for 18 hours under constant agitating. 200 µl of the supernatant were used for automated DNA extraction with the Biorobot® EZ1 (Qiagen, Germany) following the protocol of the QIAamp DNA FFPE Tissue procedure. The elution volume was 50 µl; the DNA extract was stored at −20°C. We carried out at least two independent DNA extractions and sequencings for each individual to permit authentication of the analysis results by means of comparison. Heterogeneity was only detected in ITS1 of *E. laidlawi*.

### Primer Design

Due to storage conditions and influences of the preservatives, DNA of the *E. laidlawi* specimens was degraded. Therefore, primers were designed matching the profile of ancient DNA characteristics [24] and amplifying fragments between 200 and 300 bp each (Table S2). Because of this limitation, we amplified only polymorphic sites instead of the whole genes in the cases of the mainly conserved 18 S and 28 S rRNA genes [8]. To gain information on the polymorphic sites, alignments of sequences from GenBank of numerous different species were carried out using MegAlign (Lasergene, www.DNASTAR.com) and the Clustal V algorithm. The primers were designed to discriminate against human DNA and to amplify as many taxa of Odonata as possible.

### PCR Parameters and Sequencing

The reaction volume in each setting was 25 µl, containing 12.5 µl 2x master mix (AmpliTaq® Gold 360, ABI), 0.4 µM of each primer, 5–7.5 µl of DNA extract and filled up with RNAse free water (Qiagen). PCR was carried out under the following conditions: initialization 95°C for 5 min; 40 – 45 cycles at 95°C for 1 min, *annealing temperature* (Table S2) for 1 min, 72°C for 2 min; final elongation at 72°C for 7 min; and soak at 10°C for 10 min. The PCR success and product quantity were checked by agarose gel electrophoresis. Further purification and sequencing were carried out with commercial kits (MiniElute® PCR Purification Kit, Qiagen, ABI Prism BigDye V 3.1 Terminator Cycle Sequencing Kit and NucleoSeq Kit, Macherey-Nagel) as specified by the manufacturers.

Both the forward and reverse primers used for amplification were also used for the sequencing reaction. The sequencing conditions were: initial at 96°C for 10 min; 25 cycles at 96°C for 10 s, 50°C for 5 s and 60°C for 4 min. For sequencing an ABI 310 genetic analyser with POP6 polymer was used. Sequence reads were checked for quality and assembled using SEQMAN (Lasergene; www.DNASTAR.com).

### Phylogenetic Analysis

Sequences were compiled and aligned using *MEGA* version 5 [41]. Ribosomal DNA sequences were aligned automatically with the clustalW algorithm and standard parameters. Protein coding mitochondrial sequences were aligned manually via the corresponding amino acid sequences. For alignment of ITS sequences the online version of MAFFT [27], [42] was used.

The most appropriate models of DNA substitution for bayesian and maximum likelihood tree searches were selected with MrModeltest 2.3 [43] and PAUP*4.0 b10 [44] separately for each sequence alignment and for a combined dataset containing all sequences except CO1, which was not used in the analysis since it was not possible to obtain sequences of this gene from *E. laidlawi* or *E. sinensis* specimens. The complete dataset contained 17 taxa, including a representative of Zygentoma as outgroup, and 4799 positions. Taxa from Odonata were chosen to represent all major clades as present in the phylogeny of [8]. A Nexus file of the alignments are provided as Dataset S1.

To find the most probable phylogenetic relationships of the *Epiophlebia* species the aligned sequences were combined into a mixed dataset for analysis with MrBayes 3.1.2 [45], [46]. MrBayes offers the unique possibility to reconstruct a phylogeny based on information form several different genes in a single analysis, while applying the most appropriate model to each gene sequence. Additional tree searches were done for the combined dataset with global parameters with the maximum likelihood (ML) algorithm in PAUP* and for each gene individually with MrBayes and with ML in PAUP*. To check node stability for ML a bootstrap analysis with 1000 replicates was done with the same parameters as for the original analysis.

## Supporting Information

Figure S1 **Alignment of CO2 sequences from different specimens of** ***Epiophlebia*** **species.** *E. superstes*_GB = reference sequence from GenBank.
(TIF)

Figure S2 **Alignment of ITS2 sequences from different specimens of** ***Epiophlebia*** **species.** *E. superstes*_GB = reference sequence from GenBank.
(TIF)

Table S1 **GenBank accession numbers for sequences used in phylogenetic analysis.** * = this paper.
(DOC)

Table S2 **Primers.** ^#^ this paper; * [48]; *primers in italics* are specific only for *Epiophlebia*.
(DOC)

Dataset S1 **Data matrix in NEXUS format including parameters for bayesian and maximum likelihood analysis.**
(NEX)
