# Supplementary figures and images for: Phylogeographic Analysis Elucidates the Influence of the Ice Ages on the Disjunct Distribution of Relict Dragonflies in Asia
Source: PLoS One. 2012 May 30;7(5):e38132. doi: 10.1371/journal.pone.0038132 (PMC3364219; doi:10.1371/journal.pone.0038132)

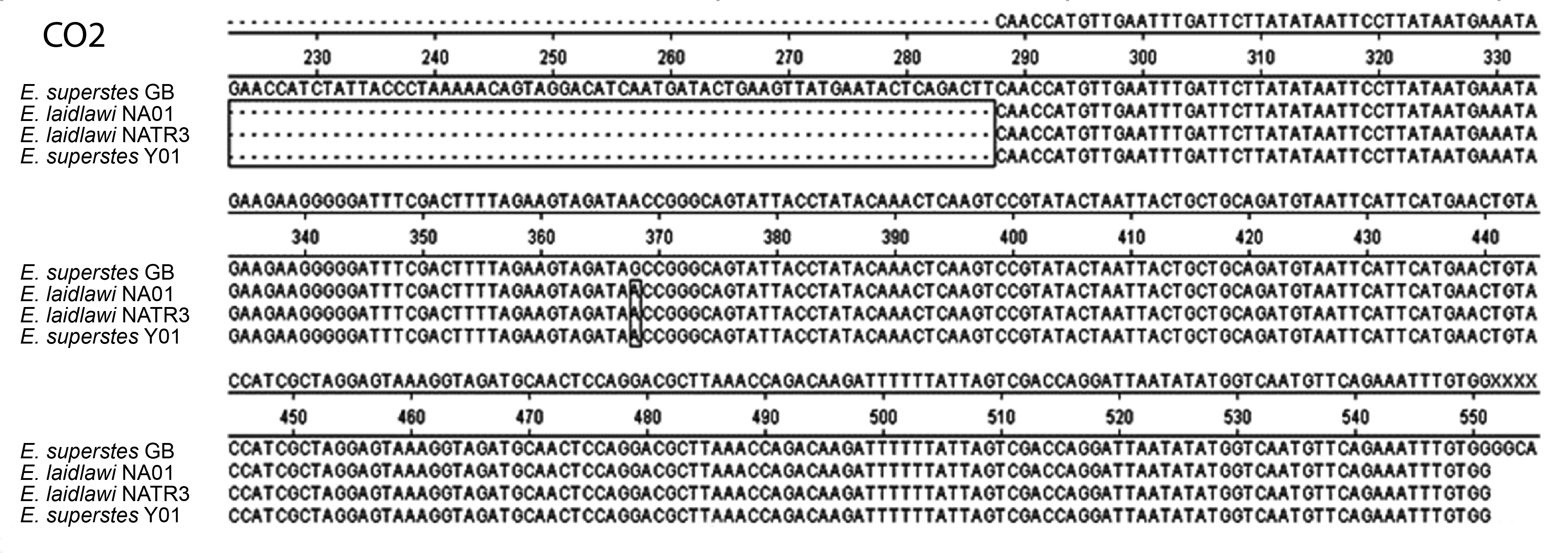

Supplement: Figure S1 — Alignment of CO2 sequences from different specimens of Epiophlebia species. E. superstes_GB = reference sequence from GenBank. (TIF) [file pone.0038132.s001.tif]

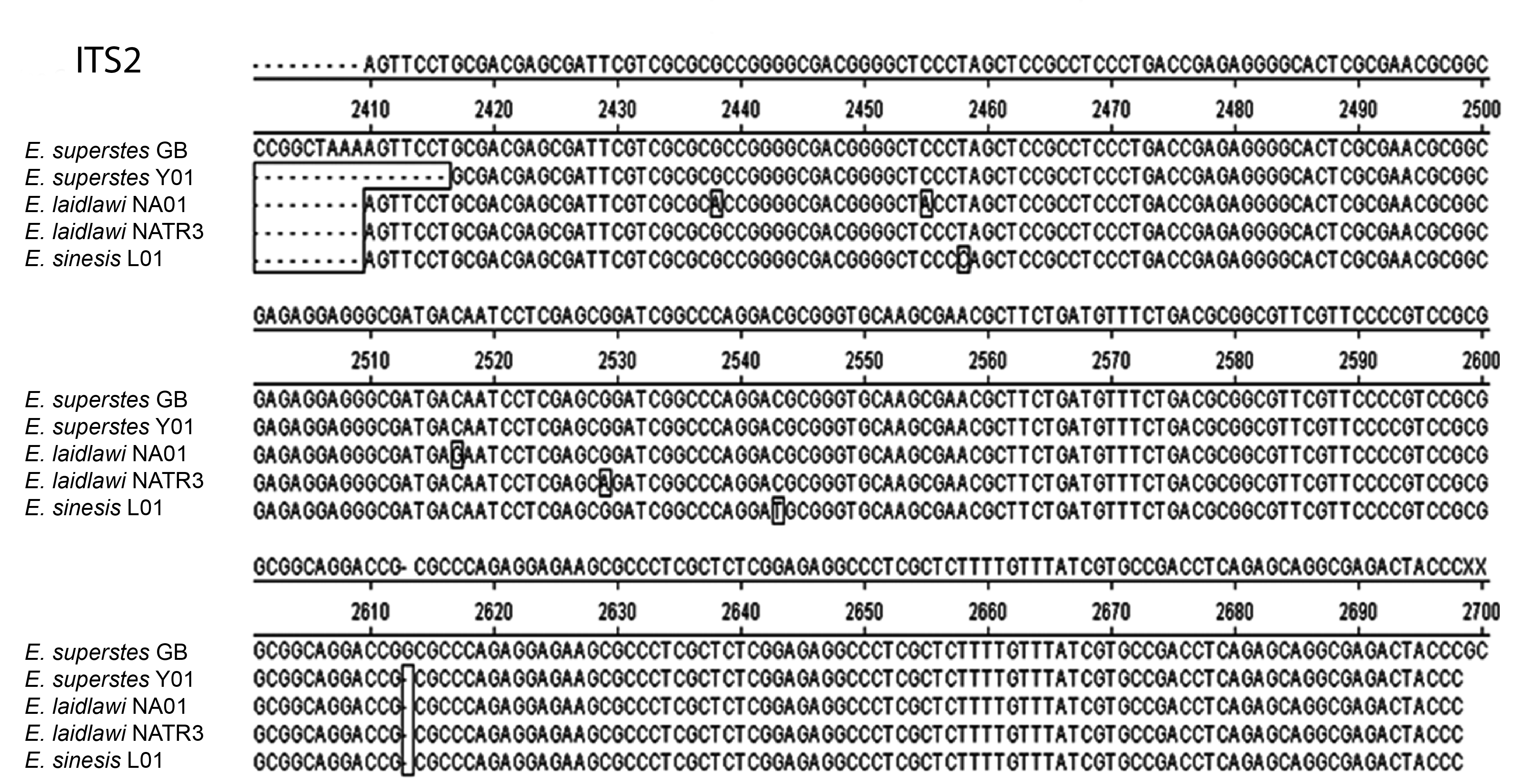

Supplement: Figure S2 — Alignment of ITS2 sequences from different specimens of Epiophlebia species. E. superstes_GB = reference sequence from GenBank. (TIF) [file pone.0038132.s002.tif]
