# Supplementary material for: Phylogeographic Analysis Elucidates the Influence of the Ice Ages on the Disjunct Distribution of Relict Dragonflies in Asia
Source: PLoS One. 2012 May 30;7(5):e38132. doi: 10.1371/journal.pone.0038132 (PMC3364219; doi:10.1371/journal.pone.0038132)
Supplement: Table S1 — GenBank accession numbers for sequences used in phylogenetic analysis. * = this paper. (DOC) [file pone.0038132.s003.doc]

**Table S1. GenBank accession numbers for sequences used in phylogenetic analysis.** * = this paper.

|  |  | **18s rDNA** | **28s rDNA** | **ITS1** | **ITS2** | **CO2** |
| --- | --- | --- | --- | --- | --- | --- |
| **Odonata: Zygoptera: Hemiphlebiidae** | *Hemiphlebia mirabilis* | FN356183 |  |  |  |  |
| **Zygentoma** | *Tricholepidion gertschi* | AF370789 |  |  |  | NC_005437 |
| **Zygentoma** | *Tricholepidion* sp. |  | AY338685 |  |  |  |
| **Zygentoma** | *Ctenolepisma longicaudata* |  |  | AY210810 | AY210810 |  |
| **Odonata: Zygoptera: Calopterygidae** | *Calopteryx aequabilis* |  |  |  |  | EU055325 |
| **Odonata: Zygoptera: Calopterygidae** | *Calopteryx splendens* | DQ008208 |  |  |  |  |
| **Odonata: Zygoptera: Calopterygidae** | *Calopteryx amata* |  | FJ009949 | AJ308361 | AJ308361 |  |
| **Odonata: Zygoptera: Platycnemidae** | *Platycnemis pennipes* | DQ008207 | FJ009928 | AJ459230 | AJ459230 | EU055397 |
| **Odonata: Zygoptera: Lestidae** | *Lestes* sp. |  |  |  |  | EU055330 |
| **Odonata: Zygoptera: Lestidae** | *Chalcolestes viridis* | AJ421949 | EU424331 | AJ421949 | AJ421949 |  |
| **Odonata: Zygoptera: Protoneuridae** | *Protoneura capillaris* | EU055184 | EU055282 |  |  | EU055381 |
| **Odonata: Zygoptera: Platystictidae** | *Palaemnema melanostigma* | FN356142 | EU055317 | FN356142 | FN356142 | EU055415 |
| **Odonata: Anisoptera: Gomphidae** | *Gomphus* sp. |  |  |  |  | EU055338 |
| **Odonata: Anisoptera: Gomphidae** | *Gomphus vulgatissimus* | FN356091 | FJ712320 | FN356091 | FN356091 |  |
| **Odonata: Anisoptera: Cordulegastridae** | *Cordulegaster dorsalis* |  |  |  |  | EU055376 |
| **Odonata: Anisoptera: Cordulegastridae** | *Cordulegaster boltonii* | FN356072 | FJ596634 | FN356072 | FN356072 |  |
| **Odonata: Anisoptera: Aeshnidae** | *Anax parthenope* |  |  |  |  | DQ166787 |
| **Odonata: Anisoptera: Aeshnidae** | *Anax imperator* | FN356035 | FJ596632 | FN356035 | FN356035 |  |
| **Odonata: Zygoptera: Pseudolestidae** | *Pseudolestes mirabilis* | EU055220 | EU055318 |  |  | FJ606784 |
| **Odonata: Anisoptera: Corduliidae** | *Hesperocordulia berthoudi* |  |  |  |  | EU055357 |
| **Odonata: Anisoptera: Corduliidae** | *Idionyx imbricata* | EU055189 | EU055287 |  |  |  |
| **Odonata: Anisoptera: Corduliidae** | *Idionyx optata* |  |  | FN356096 | FN356096 |  |
| **Odonata: Zygoptera: Coenagrionidae** | *Ischnura elegans* |  |  | FN356103 | FN356103 | HQ834805 |
| **Odonata: Zygoptera: Coenagrionidae** | *Ischnura barberi* | EU055136 | EU055231 |  |  |  |
| **Odonata: Zygoptera: Heliocharitidae** | *Heliocharis amazona* | AJ746326 |  | AJ746326 | AJ746326 |  |
| **Odonata: "Anisozygoptera"** | *Epiophlebia superstes* | FN356086 | EU424328 | FN356086 | FN356086 | EU055421 |
| **Odonata: "Anisozygoptera"** | *Epiophlebia superstes** | JQ943599 | JQ943588 (S1), JQ943589 (S2), JQ943590 (S3), JQ943591 (S4 | JQ943606 | JQ943609 | JQ943603 |
| **Odonata: "Anisozygoptera"** | *Epiophlebia laidlawi** NATR3 | JQ943597 |  |  | JQ943611 | JQ943605 |
| **Odonata: "Anisozygoptera"** | *Epiophlebia laidlawi** NA01 | JQ943598 | JQ943592 (S1), JQ943593 (S2), JQ943594 (S3), JQ943595 (S4) | JQ943607 | JQ943610 | JQ943604 |
| **Odonata: "Anisozygoptera"** | *Epiophlebia sinensis** |  |  | JQ943608 |  |  |
