# Supplementary material for: Phylogeographic Analysis Elucidates the Influence of the Ice Ages on the Disjunct Distribution of Relict Dragonflies in Asia
Source: PLoS One. 2012 May 30;7(5):e38132. doi: 10.1371/journal.pone.0038132 (PMC3364219; doi:10.1371/journal.pone.0038132)
Supplement: Table S2 — Primers. # this paper; * [48]; primers in italics are specific only for Epiophlebia. (DOC) [file pone.0038132.s004.doc]

**Table S2. Primers.**

| System | Primer | Sequence (5`-3´) | Length of resulting sequence(bp) | Annealing temperature |
| --- | --- | --- | --- | --- |
| 18S rDNA | 18S_up^#^ | GGTTCCTTGGATCTTACCCACACT | 240 | 59°C |
|  | 18S_low^#^ | GCAGAACCTACCATCGAAAGTTGAT |  |  |
| 28S rDNA-1 | 28S_up^#^ | TCGGACACGCTCCGCTAAAC | 191 | 64°C |
|  | 28S_low^#^ | GCCAGGCATAGTTCACCATCTTTC |  |  |
| 28S rDNA-2 | 28S2_up^#^ | CCGGTAAAGCGAATGATTAGAG | 267 | 60°C |
|  | 28S2_low^#^ | CCACCGTCCTGCTGTCTTAA |  |  |
| 28S rDNA-3 | 28S3_up^#^ | GGAATCCGCTAAGGAGTGTGTAA | 251 | 58°C |
|  | 28S3_low^#^ | AGGGCCTCGCTGGAGTATTT |  |  |
| 28S rDNA-4 | 28S4_up^#^ | CCGTTGCACACGAGTCAGTC | 293 | 58°C |
|  | 28S4_low^#^ | TCGCGTTCCAAACCCTATCT |  |  |
| *ITS1* | *ITS1_up* | *CCTGCGGAAGGATCATTATTGT* | *215* | *56°C* |
|  | *ITS1_low* | *GGTGGGTTCAGGGGAGAGAC* |  |  |
| *ITS2* | *ITS2_up* | *AGTTCCTGCGACGAGCGATT* | *289* | *62°C* |
|  | *ITS2_low* | *GGGTAGTCTCGCCTGCTCTGA* |  |  |
| CO2 | CO2_up | TCAACCATGTTGAATTTGATTCTTAT | 265 | 55°C |
|  | CO2_low | CCACAAATTTCTGAACATTGACC |  |  |
| CO1 | CO1_up* | GGATCACCTGATATAGCATTCCC | 500 | 50°C |
|  | CO1_low* | CCCGGTAAAATTAAAATATAAACTTC |  |  |

^#^ this paper; *[40]; *primers in italics* are specific only for *Epiophlebia*.
